# Commentary: Efficacy and Safety of Dupilumab in Moderate-to-Severe Bullous Pemphigoid

**DOI:** 10.3389/fimmu.2021.800609

**Published:** 2021-11-23

**Authors:** Si-Hang Wang, Ya-Gang Zuo

**Affiliations:** Department of Dermatology, State Key Laboratory of Complex Severe and Rare Diseases, Peking Union Medical College Hospital, Chinese Academy of Medical Science and Peking Union Medical College, National Clinical Research Center for Dermatologic and Immunologic Diseases, Beijing, China

**Keywords:** bullous pemphigoid, dupilumab, treatment, IL-4, IL-13

## Introduction

Bullous pemphigoid (BP), a chronic autoimmune cutaneous blistering disorder affecting predominantly the elderly, is characterized by skin tense bullae formation and pruritus symptoms (1). Current treatments for mild BP patients include topical high potency corticosteroids, anti-inflammatory antibiotics (minocycline or doxycycline), and nicotinamide. Despite its toxicity and relatively high prevalence of adverse events, systemic nonspecific immunosuppressive therapies are applied for moderate to severe BP. Given the significant adverse effect caused by systemic steroids, novel targeted therapeutic approaches are warranted to provide corticosteroid-sparing with a favorable safety profile and induce durable drug responses. Additionally, traditional Chinese medicine, Tripterygium wilfordii Hook F (TwHF), in the treatment of BP is widespread in China on its immunomodulatory and anti-inflammatory properties. A low dose of 30-60 mg daily of TwHF is very well-tolerated and efficacious in mild or moderate BP patients (2). TwHF has been wildly used in our department as a corticosteroid-sparing agent, demonstrating a better safety profile than prednisolone monotherapy (2).

The improving awareness of the immunopathogenesis of BP has sparked an interest in newly-developed biologics as potentially efficient and safer alternative therapeutic options. Targeted therapies inhibiting T helper 2 (Th2) responses and its downstream immune cascade is appealing as increased evidence suggested their involvements in BP pathogenesis. Omalizumab targeting IgE, bertilimumab inactivating eotaxin-1, and mepolizumab blocking interleukin (IL)-5 have all recently been tested or applied in BP (3). In this respect, Zhang et al. recently described the efficacy and safety of dupilumab, an IL-4 receptor alpha unit antagonist inhibiting both IL-4 and IL-13 functions, in combination with conventional therapies (methylprednisolone and azathioprine) *versus* conventional therapies alone for treatment of 24 cases of moderate to severe BP (4). Time to stop new blisters formation and the total amount of conventional systemic therapies were significantly reduced in the dupilumab group, with a potent itching relieving activity showing in patients treated with dupilumab. More importantly, dupilumab introduction largely facilitated the tapering course of methylprednisolone, leading to disease control in a shorter time and a lower recurrence rate in a 32 weeks follow-up. This is the first retrospective case-control study in dupilumab treatment for BP.

**Figure 1:**
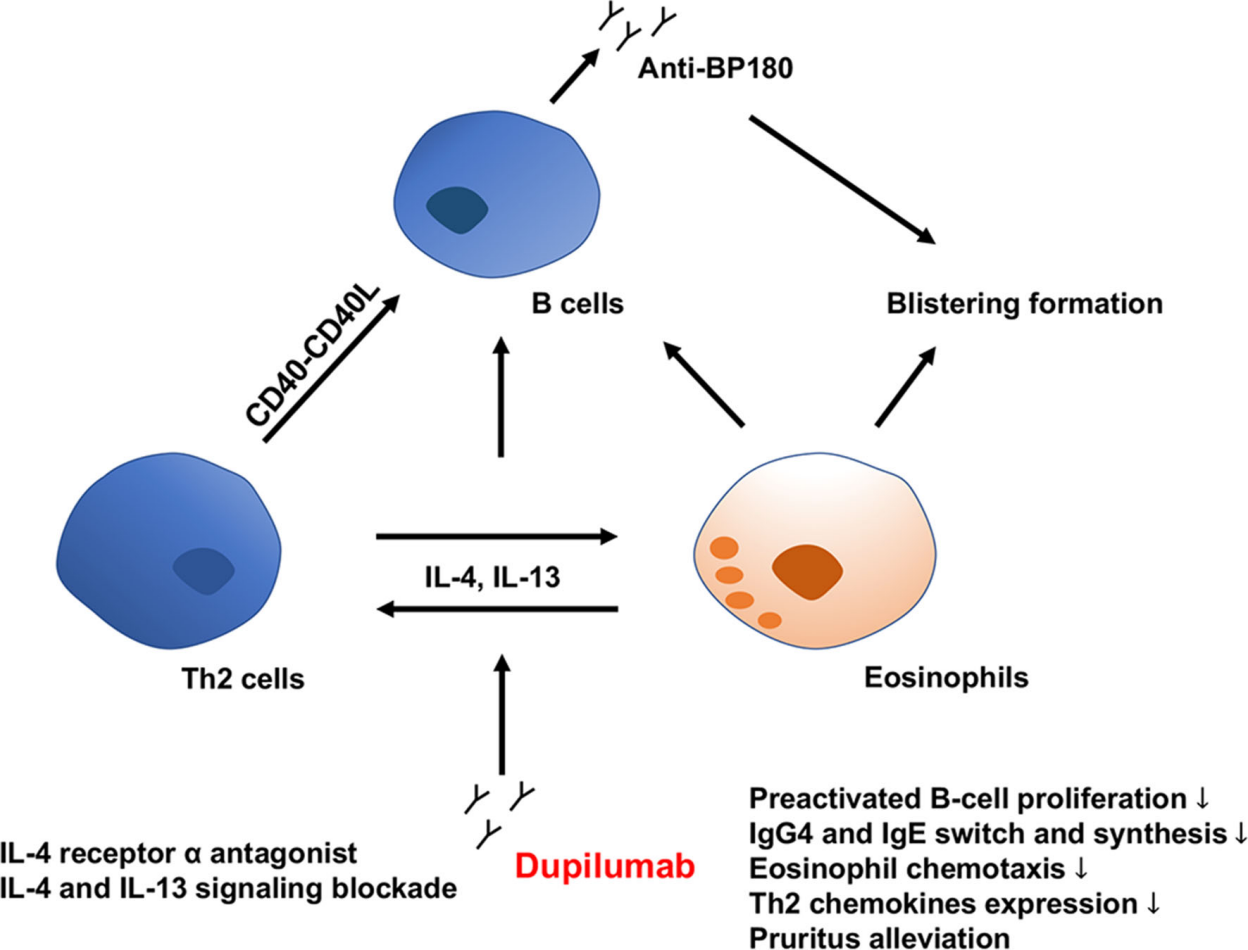
Direct and indirect activities of dupilumab in the immunopathogenesis of BP. BP, bullous pemphigoid; IL, interleukin; Th2, type 2 T helper; Ig, immunoglobin.

Complicated immune interactions concerning the Th2 axis are markedly involved in BP pathogenesis (**Figure 1**). Th2 cells are strongly associated with the loss of tolerance of BP180 antigen in BP. Th2 cell epitope peptides, P2 (492-506 aa: VRKLKARVDELERIR) and P5 (501-515 aa: ELERIRRSILPYGDS) from BP180 NC16A domain contributed to CD4+T cell activation, IL-4 expression, and B cells autoantibodies production in BP patients (5). Th2-associated cytokines (IL-4, IL-5, IL-13 and IL-31), and Th2-related chemokines (eotaxin, chemokine C-C motif ligand [CCL]13, and CCL18), as well as IgE and eosinophils, have been demonstrated to be significantly involved in the BP development (6–9). IL-4 and IL-13 are relevant to the recruitment and adhesion of eosinophil, differentiation of B lymphocytes, as well as the switch and synthesis of IgG4/IgE antibodies (7, 10–12). Levels of Th2-related chemokines CCL18 are also markedly higher in the blister fluids than in the sera of BP patients and control group. Furthermore, these elevated expressions correlate immediately with the disease course (13). In addition to causing dermal-epidermal separation and blistering formation, eosinophils may perpetuate Th2 inflammation by secretion of IL-4, IL-5, and IL-13, indicating an eosinophil-Th2 cell interaction in BP (9). Also, eosinophils and cytokines such as IL-13 are closely connected with BP-related itching (14). Compared with healthy individuals, IL-4 and IL-13-expressing T cells were elevated both in the sera and blisters of BP patients, showing a gradient between blister fluid and sera levels. After treatment with corticosteroids systemically, skin-homing IL-13 expressing T cells were significantly decreased, as well as an apparent clinical improvement, suggestive of a critical activity of IL-13 in the pathogenesis of BP lesions (11). One study detected the cytokine response to BP180 NC16A recombinant and proved a characteristic IgE and IL-4 effect with the strongest association with BP, providing evidence that IL-4 contributes significantly to BP pathogenesis (12). Given the vital role of IL-13 and IL-4 in BP development, inhibiting both of Th2 cytokines may result in a new therapeutic alternative in BP treatment.

Over the past five years, a variety of small case reports and series have described the efficacy of dupilumab in BP (15–19), in which a multicenter case series of 13 refractory BP patients was conducted in American, leading to satisfactory disease control in most cases (15). No dupilumab-related adverse events reported thus far. In clinical practice, similar efficacy was observed in our department when recalcitrant BP was treated with dupilumab combined with conventional therapies methylprednisolone and TwHF. Nevertheless, in some cases, transient eosinophilia was observed in dupilumab monotherapy (unpublished data), which may be attributed to the unblocked Th2 cytokine IL-5 mediated eosinophil released from bone marrow. Concomitant non-specific immunosuppressive therapies in dupilumab group may account for absent of eosinophilia in this study.

However, some concerns should be focused on. The medical history of 24 BP patients was not described in this study. Patients who take medications known to cause or aggravate BP (for example, angiotensin-converting enzyme inhibitors and dipeptidyl peptidase-4 inhibitors) and have other complications (for the possibility of tumor-associated BP) should be excluded or described clearly in methods. Also, the immunological effects of dupilumab such as anti-BP180 and anti-BP230 antibody titers and Th2-related cytokines/chemokines at pre-and post-treatment stages have not been elucidated. Additionally, the initial dosing regimen of concomitant systemic corticosteroids in the study was methylprednisolone 0.6 mg/kg/day for moderate and severe patients. According to the international guideline of BP, prednisolone 0.5 mg/kg/d and 0.75-1 mg/kg/d (methylprednisolone 0.4 mg/kg/d and 0.6-0.8 mg/kg/d) for moderate and severe patients, respectively, are maximally tolerable and efficacious (1). The study design should distinguish the different severity of BP receiving appropriate systemic steroids. Although increasing evidence suggests dupilumab treatment in recalcitrant BP, further prospective randomized controlled clinical trials with larger samples and more extended follow-up period are needed to clarify the optimized dosage and interval of dupilumab and define appropriate BP patient selection criteria.

## Author Contributions

The manuscript was written by S-HW with significant contributions from Y-GZ. All authors contributed to the article and approved the submitted version.

## Funding

This study was supported by the National Natural Science Foundation of China (grant number 81972944) and the Beijing Natural Science Foundation (grant number 7192166).

## Conflict of Interest

The authors declare that the research was conducted in the absence of any commercial or financial relationships that could be construed as a potential conflict of interest.

## Publisher’s Note

All claims expressed in this article are solely those of the authors and do not necessarily represent those of their affiliated organizations, or those of the publisher, the editors and the reviewers. Any product that may be evaluated in this article, or claim that may be made by its manufacturer, is not guaranteed or endorsed by the publisher.
